# “I Couldn’t Have Asked for a Better Quarantine Partner!”: Experiences with Companion Dogs during Covid-19

**DOI:** 10.3390/ani11020330

**Published:** 2021-01-28

**Authors:** Cori Bussolari, Jennifer Currin-McCulloch, Wendy Packman, Lori Kogan, Phyllis Erdman

**Affiliations:** 1 Counseling Psychology, University of San Francisco, San Francisco, CA 94117, USA; 2 School of Social Work, Colorado State University, Fort Collins, CO 80523, USA; jen.currin-mcculloch@colostate.edu; 3 Department of Psychology, Palo Alto University, Palo Alto, CA 94304, USA; wpackman@paloaltou.edu; 4 Clinical Sciences, Colorado State University, Fort Collins, CO 80523, USA; lori.kogan@colostate.edu; 5 College of Education, Washington State University, Pullman, WA 99163, USA; perdman@wsu.edu

**Keywords:** Covid-19, companion–animal relationships, human–dog bond, social isolation, dog ownership

## Abstract

**Simple Summary:**

The purpose of this qualitative study was to examine how Covid-19 restrictions influenced dog owners’ relationships and sense of connection with their canine companions. Data were collected through an on-line survey and themes from open-ended questions were coded by the researchers using directed content analysis. Results highlighted a strong human–animal appreciation, and that dog ownership during this pandemic diminished participants’ sense of isolation and loneliness, as well as supported their mental/physical health.

**Abstract:**

The Covid-19 pandemic has been found to negatively impact the psychological well-being of significant numbers of people globally. Many individuals have been challenged by social distancing mandates and the resultant social isolation. Humans, in our modern world, have rarely been as isolated and socially restricted. Social connectedness and support are critical protective factors for human survival and well-being. Social isolation can lead to loneliness, boredom, and can become a risk factor for physical and mental health issues such as anxiety and depression. The attachments formed with dogs, however, can be as strong or even stronger than human connections, and has been shown to relate to fewer physical health and mental health problems, as well as decrease isolation and loneliness. The purpose of this qualitative research was to examine the thoughts, experiences and concerns of 4105 adults regarding their companion dog during the initial months of Covid-19. Data were collected between March 31st–April 19th, 2020 via online survey and themes were coded by the researchers using directed content analysis. Results highlighted a strong human–animal appreciation, and that dog ownership during this pandemic diminished participants’ sense of isolation and loneliness, as well as supported their mental/physical health.

## 1. Introduction

The Covid-19 pandemic has impacted people’s lives in profound ways. Many individuals have been affected by financial/work losses, yet perhaps even more have been challenged by social distancing mandates and the resultant social isolation. Most humans, in our modern world, have rarely been as isolated and socially restricted. Countless individuals, due to social distancing and stay-at-home mandates accompanying the initial stages of Covid-19 were physically separated from friends and family. For many people during this time, mobile phones and online platforms replaced in-person contact as a primary source of social connection and support [1,2,3]. The importance of this isolation cannot be overstated; social connectedness and support are critical protective factors for human survival and well-being [4]. Social isolation can lead to loneliness, boredom, and in prolonged cases, can become a risk factor for physical [5,6] and mental health issues such as anxiety and depression [3,7]. It is for these reasons that the psychosocial impacts of a pandemic, as experienced during Covid-19, may be more far-reaching than the virus itself. In contrast to the biological effects of the virus on the global population, the psychosocial effects of the pandemic are “more pronounced, more widespread, and longer lasting than the purely somatic effects of infection” (p. 23) [2].

In addition to social isolation, a great amount of uncertainty accompanies a pandemic, with a tolerance of uncertainty playing a significant role in one’s experiences of anxiety, distress, and loss of control [2,8,9]. Pandemics are “marked by uncertainty, confusion, and a sense of urgency” p. 25 [2] and can cause individuals to experience considerable distress, avoid others, and impair the ability to adequately function [10]. As Banerjee and Rai [11] so adeptly stated:

“Humankind has always known what to do next, with their lives generally following a regular trail. But this sudden cataclysmic turn of events (Covid-19) has brought them face to face with a dire reckoning—how to live with oneself. It is indeed a frightening realization when a whole generation or two knows how to deal with a nuclear fallout but are at their wit’s end on how to spend time with oneself (p. 525).”

Recent studies suggest that the pandemic negatively impacted the psychological well-being of significant numbers of people. A US study by Palsson, Ballou, and Gray [12] found 90% of survey respondents reported experiencing emotional distress related to the pandemic. Moreover, when respondents were asked to compare their current stress levels (in May 2020) to the month of January 2020 (one month before Covid-19 threat), 55% of respondents reported feeling that their life had become more stressful. These negative emotional reactions appear to be universal. The results from a study that assessed on-line Chinese social media usage found an increase in negative emotions (i.e., depression, anxiety) and a decrease in positive emotions and life satisfaction when compared to pre-Covid times [13]. Other researchers in China found that over half of the respondents noted the psychological impact of Covid-19 as moderate to severe [14], and a third study reported increased levels of numerous psychological problems as a result of strict quarantine measures [15]. Studies from other countries report similar trends [16,17]. As people struggle to maintain social connections and a sense of certainty, many may look to their dogs, companions that have been found to offer a myriad of benefits for humans. [18]

### Benefits of the Human–Dog Relationship

A plethora of research supports the premise of a strong bond between humans and their companion dogs [19,20,21]. Dogs provide unconditional and non-judgmental love [22] and dog companionship is related to fewer physical health and mental health problems [23,24]. Owning a dog has also been shown to decrease isolation and loneliness [25] as well as provide support during stressful times [26]. For some people, the attachments formed with dogs can be as strong or even stronger than human connections. Indeed, it is increasingly clear that humans bond with nonhuman animals in ways that are comparable with human–human attachment [27]. In fact, interactions with dogs have been shown to increase levels of oxytocin in humans, paralleling oxytocin effects seen among mothers breastfeeding infants [28]. It is not surprising, therefore, that dogs are often seen as part of the family [27] or even as surrogate children [29]. For many people, the relationship they have with their dog is one of their most significant connections, and as a result, the loss of this relationship when a dog dies is often profound [30,31,32].

In 2017, there were reportedly 90 million owned dogs in the United States [18]. Given the enormous number of dog owners affected by the pandemic, and our current understanding of the formidable human and companion dog bond, we were interested in asking participants to share their thoughts, experiences, and concerns regarding their companion dog during the initial months of Covid-19.

## 2. Materials and Methods 

### 2.1. Participants and Procedure

Dog owners were recruited via requests posted online and direct personal solicitations to the survey entitled “Pet dogs during the time of Covid-19”. A cover letter explaining the goal of the study, the affiliation of researchers, and a link to the Qualtrics survey website was sent to potential participants. Eligible participants were required to be at least 18 years of age, from any country, and must have been living with a companion dog during the time of the Covid-19 pandemic. The study was approved by the Institutional Review Board at Colorado State University (20-10003H). Survey respondents were recruited through social media outlets and human animal focused organizations (e.g., Facebook dog focused groups, Human Animal Interaction Section of American Psychological Association, etc.). Participants completed the anonymous survey on the Internet.

At the time of data analysis, there were 4105 respondents to the survey (received between 31 March–19 April 2020). Respondents included a majority of females (88.3%) living in the United States (80.7%), and those between ages 50–59 (23.8%). The majority of respondents lived with one other adult in the home (61.7%), had no children living at home (80.5%) and lived within a community that had implemented significant social interaction restrictions (78.0%) (See Table 1).

To capture the unique experiences of living with a companion dog during the time of a pandemic, the research team created a novel survey tool. In addition to sociodemographic variables, the survey inquired about participants’ levels of social restriction, and access to social support, dog food, and medical care. Survey items then delved into coping status and the human–animal bond. The survey consisted of Likert-scale items (not included in this analysis) and four open-ended questions that invited participants to share their thoughts about pandemic-related changes in their relationships with their dogs, stressors, and other ways in which Covid-19 impacted their relationships or feelings about living with their dog.

Due to the large number of responses, a representative sample, using a systematic sampling method, was selected for each of the four open-ended questions. The initial sampling step included cleaning the data and removing of fields with no qualitative responses. Next, researchers utilized a random number generator in Excel to randomly sort participants on each of the four questions. Finally, the first 10% (N = 410) of the randomized responses for each open-ended question were chosen for analysis. Thus, respondents were not pre-examined to “cherry-pick.” As long as the starting point is randomized, systematic sampling is considered to be a type of probability sampling for selecting a random sample [33].

### 2.2. Research Design: Qualitative Analytic Procedure

The researchers exported all responses to the qualitative questions into an Excel file. Each row of the file was a different respondent, and each column was a question of the survey the participants answered. Within the Excel file, four separate tabs/folders were created to individually hold the 10% of culled data for each of the four open-ended questions. Next, data from each of the Excel tabs were pulled and saved into individual Microsoft Word tables, one for each qualitative question. The first column of the table included each participant’s response to the question and the subsequent columns provided space for the coding data from that individual’s response. The researchers saved the data to secure individual computers for analysis in Microsoft Word.

Researchers used directed content analysis, a qualitative method guided by theory or prior research [34], to analyze participants’ responses to each question. “The goal of a directed approach to content analysis is to validate or extend conceptually a theoretical framework or theory” (p. 1278) [35]. Content analysis using such an approach is a more structured process than conventional content analysis [36]. Investigators begin by identifying key concepts as initial coding categories [34]. Next, operational definitions for each coding category were determined based on theory. In the current investigation, human-animal interaction/pet attachment and continuing bonds theories [37,38] as well as research on positive emotions [39] and pandemics [9] guided the development of initial coding categories. Data that could not be coded were identified and analyzed later to determine if they represented a new theme or a subcategory of an existing category. The major strength of directed content analysis is that “existing theory can be supported and extended” (p. 1283) (35).

The responses were first independently coded by all three authors and then reviewed by all three until consensus was reached and tracked in a master codebook. As noted above, the emerging themes were identified and categorized and new codes developed as needed. In addition to the coding, networks were created to assist in the analysis of themes among subgroups of participants.

The research team kept an audit trail throughout data analysis to track emerging themes, code definitions, as well as code and meaning saturation [40]. As new themes appeared in the data, the team marked the emergence of each theme in the master codebook. The coders also discussed coding trends among the team, patterns among respondents, and nuanced meanings. The team also monitored for saturation and noted within the master codebook the point in which new data no longer elicited new codes (code saturation) or flushed out new meanings for the codes (meaning saturation). This procedure was followed for each of the four questions.

## 3. Results

Each of the four open-ended questions resulted in different themes. The presentation of results includes a synopsis of responses for each question and introduces the most prevalent themes and how they related to participants’ experiences in coping during the pandemic.

### 3.1. Having a Dog at Home: Impact on Stress Level

Q1. There are many stressors that can come with Covid-19. Do you feel that having a dog adds, reduces, or has no impact on your stress level?

Given the onslaught of changes and uncertainty resulting from Covid-19, we were concerned how quarantine orders limited access to resources and fears about one’s own health and what may happen in the future would impact participants. Thus, we asked if participants felt that having a dog reduces, adds, or has no impact on their stress level, and to elaborate on their responses. The majority of respondents (76.8%) stated that having a dog reduced their level of distress, while a few individuals (6.4%) shared that their dog added stress during the pandemic. A small number (12.0%) felt that their dog had no impact on their stress level. The three most common themes in participants’ responses to this question all related to ways in which their dog reduce their stress: displaces worry/serves as a distraction from Covid-19 (14.0%); diminishes feelings of isolation and loneliness (13.2%); and improves their mental health (11.0%).

#### 3.1.1. Reduces Stress

##### Displaces Worry and Distracts from Covid-19

The majority of respondents stated that having a canine companion ameliorated their stress related to the uncertainty, loss, and grief associated with Covid-19. The most common response among participants was the way in which their dogs helped to displace their worry and distract them from the everyday challenges resulting from Covid-19. A respondent echoed the sentiment of others that caring for their dog “Overall lowers my stress level, as I have to concentrate on caring for him rather than being anxious about myself.” Dogs’ playful spirits also fostered joy, silliness, and opportunities to laugh, when laughter often felt silenced by the pandemic. For example, “My dog does not know what is going on in the world, she does her happy doggy-things and takes me with her in a moment where there are no worries, just joy”. As individuals adjusted to new schedules and increased time at home, caring for dogs provided a semblance of structure. Daily walking and feeding routines also nurtured a sense of normalcy during the surreal state of the pandemic. One participant explained, “Having a being and tasks and activities to do that are about something other than Covid related, good distraction, something that remains normal”. Canine companions nourished their humans’ abilities to find moments of distraction from the pandemic and opportunities to explore purpose and meaning when many of their identities and roles were threatened by the magnitude of loss and uncertainty resulting from Covid-19.

##### Diminished Respondents’ Overwhelming Sense of Isolation and Loneliness

Having a dog diminished respondents’ overwhelming sense of isolation and loneliness and provided a sense of connection with another living being and the outside world. Due to social distancing restrictions, canine companions often filled central roles in social support systems, especially for individuals living alone during the lock-down stages of the pandemic. Many individuals felt isolated from their former worlds and shared the following sentiments: “Companionship is necessary. (My dog) is a member of our family and we are able to interact, talk, touch, etc. with her. Knowing that she enjoys our company is nice too”. Often, dogs derive great comfort in the company of their humans and express these sentiments through cuddles, kisses, and snuggles. These moments of affection nurture humans’ desires for physical touch. Participants provided accolades for their dogs: “Having a dog reduces my stress as it creates predictable routines and a living being I can interact with when I am socially distancing from others. Additionally, my pet helps me connect with nature and is entertaining and loving”. Expressions of the love and comfort they derived from their dogs were plentiful in these owners’ descriptions of the positive presence their dogs had in their lives.

##### Mental and Physical Health Benefits

Living with and caring for a dog brought many secondary mental and physical health benefits to these owners. Since many of these individuals, due to Covid restrictions, were at home much more, the amount of time they spent with their dogs naturally increased. Subsequently, humans had more time to exercise, play, and engage in fun activities with their dogs. Having this new-found bonding time with dogs, “reduces the impact (of Covid), because staying at home with your dog is relaxing, you are exercising and giving love, you are not just thinking about the problems you are having in relation to Covid-19, and it improves your mental and physical health”. Several respondents commented on the health benefits of increased walks with their canine companions: “definitely decreases stress by walking them every day now I keep myself saner and get exercise”. More time playing and exercising with dogs also created work–life balance and time away from human co-quarantine partners, “It forces me to go outside and get fresh air and I have support and company when quarantined with difficult family”. Increased time with canine companions encouraged more physical activities and acted as a buffer from work, relationships, and other pandemic-related stressors.

#### 3.1.2. Adds Stress

Albeit a small percentage of overall responses, there were three themes that represented the ways in which having a dog during the pandemic increased respondents’ stress: worries about the future (10.1%), negotiating multiple roles and responsibilities (5.1%), and dog behaviors (3.7%). The quick and deadly spread of the virus and persistent scarcity of many necessities (for people and canines alike) created grave concerns among some participants. Many respondents, especially those who lived alone or were frail, indicated that they were worried about what might happen to their dogs in the event that the respondent contracted Covid-19. They worried about who would provide daily care for their dog should they enter the hospital. Worry presented in the following way:

“(My dog) adds (stress) b/c I’m worried about leaving him with others if I need to be hospitalized with C-19. He’s a senior with kidney disease and on his last leg and I fear for his reaction to the changes in his life not only in the short run but also if I die in hospital as I’m a senior.”

Through the development of safety plans for their dogs, some people were able to ameliorate their stress:

“I live alone and I’ve been worried about what happens to him if I get sick, so that’s been a stressor but I sat down & wrote out instructions for him & just put all his general info down on paper just in case something happens to me and that has significantly reduced the stress with that issue.”

Other persistent worries included fear of not being able to access or afford emergency veterinary medical care and specialty dog foods.

##### Negotiating Multiple Roles and Responsibilities

Transitions from offsite work settings to home offices resulted in numerous stressors for individuals as they attempted to negotiate multiple roles and responsibilities. Individuals had heightened anxieties about how their virtual work world would unfold. They felt competing needs to manage Zoom work meetings while also caring for the emotional and social needs of their dogs. One person explained, “(My dog) adds to the stress because he expects more attention with me being home more”. This added stress also appeared as personal remorse, “like guilt that I’m home but have to work so I can’t spend that time playing”. Due to public health mandates, many of the typical activities that dogs enjoyed as social engagement and stress releases became unavailable. Dogs could no longer go to doggy daycare, dog parks, or participate in service dog activities. As a result, dog behaviors weighed heavier on individuals than before the pandemic. This became most apparent in responses of those that had puppies, “(He) adds stress as he is 1 and still learning what he’s not allowed to do”. Many folks carried an emotional burden of feeling like they were not able to provide for their dog’s social and mental stimulation. One respondent described this added stress, “We can’t leave the house and she’s so unhappy which makes me unhappy. I’m failing her”. Although outside of their control, activity restrictions created an additional layer of stress.

#### 3.1.3. No Impact

Some individuals responded that being a dog guardian during Covid-19 has not impacted or not added additional stress to their daily lives. A few individuals simply responded “no impact”, while others described the buffering effects of having stable access to financial resources, dog walkers, and veterinary care. Respondents in rural areas not yet affected by the pandemic typically described no change in their level of stress: “I am fortunate enough that our situation is minimally impacted by Covid-19 at this time. We live in a remote community, and work from home”. Those who already worked from home reported limited impact on their stress since their daily routines with their dogs remained similar. Public health information demonstrating no possibility for spread of contagion between humans and dogs was included as another reason for not experiencing impact on stress levels.

Responses occasionally included a mixture of both adding and reducing stress, underscoring the complexity of how the initial stages of the pandemic affected dog owners; subsequently, these responses were coded for the added stressors, as well as the ways that their dog reduced their stress. For example:

On the one hand this situation of emergency and uncertainty increases anxiety about the future and the conditions of future life namely economic and financial risks and the ability to care for my dog and cats with the same availability and quality. On the other hand, the relationship I have with them helps to reassure me and to focus on the emotional and organic relational stability that we maintain and which is so essential. 

Overall, participants extolled the benefits of having a dog during the pandemic as their canine companions created a sense of connection, a calming presence, and a distraction from the news and the wrath of Covid-19.

### 3.2. Impact of Increased Time Spent with Dog

Q2. Do you feel the increased amount of time you are spending with your dog is strengthening your relationship or creating strain in the relationship?

The aggressive spread of Covid-19 resulted in public health precautions which limited social contact. Individuals in jobs that were not identified as essential to health and service industries shifted from office settings to home settings. Learning in schools moved from in-person to online home education. The increased time at home created opportunities to both strengthen or strain bonds between individuals and their dogs. The majority of respondents (76.8%) claimed that spending more time with their canine companions strengthened their relationships, while a few individuals (3.2%) shared that increased time together placed more strain on their relationship. Several individuals (20%) felt the increased time together both strengthened and strained their relationship with their dog.

#### 3.2.1. Strengthens

The three most common themes all related to ways in which the increased time spent with their dog strengthened their relationships: more quality time; increased attunement/intuition; and increased playtime and training time.

##### More Quality Time

Many respondents stated that the supplemental time with their companion dogs strengthened their relationship. Participants described their time with their dogs prior to Covid as limited. Due to Covid, they had more time to share with their dogs and could take advantage of more hugs, cuddles, and relaxing time together. Working from home offered “loads of flexibility and spending more time focusing on what they need versus the way it was before Covid when I was always in a hurry”. Increased time appeared mutually beneficial for humans and their canine companions. Participants’ responses indicated they felt their dogs were happier, as well as more joyful and affectionate: “He never leaves my side. He’s happy when I’m around and I get lots of love and furry therapy from him”. Those caring for elderly and frail dogs savored additional precious moments with their dogs: “My dog is basically on hospice and can deteriorate at any time prompting euthanasia. I am pleased to be able to spend this time with him to look after him, provide good care and improve his morale”. In their roles as caregivers, participants cherished the additional quality time to nurture the affection, love, and companionship they shared with their canine companion.

##### Increased Attunement/Intuition

Spending more time together resulted in participants expressing increased attunement/intuition with their canine companions (13.7%). Not only did respondents experience an increased understanding of their dog’s needs and emotions; they also felt that their dogs became more attuned to changes in their stress levels. This bi-directional attunement was described as an enhanced ability to read each other’s body language and communicate. Statements of dogs’ increased intuition echoed throughout responses: “Yes, he definitely senses there are major changes and he can sense my children’s anxiety. He tries to be there for everyone. Offering his free petting services.” Given limitations on social contact, canine companions provided emotional connections previously filled by humans. Participants reported that they felt that their dogs intuitively knew when to appear for hugs or to bring moments of laughter and joy. Humans also expressed experiencing a greater sense of trust from their dogs. For instance, “My dog relies on me more now that I am home more often. To me it’s a sign of greater trust”.

##### Increased Playtime and Training Time

As participants navigated social distance restrictions and more time at home, many chose to spend their new-found time training and/or playing with their dog. While the closures of dog training facilities, doggy day cares, and dog parks limited the typical structured activities used to train and entertain dogs, daily walks and training activities increased. These included walking on leashes, improving basic obedience, reducing barking, and rough-housing with other animals or small children in the house. Families with puppies portrayed the urgent need to work on puppy behaviors and obedience. Noticeable changes in dogs’ behaviors materialized: “I spend more time walking and training her. She has increased clarity on what I want because of the increased training time”. Playtimes centered on games in the yard and inside the house, going for walks, chasing toys, and interacting with other animals and kids at home. This increased playtime nurtured family bonds: “My dog is so happy to have the entire family home and is going on lots of walks and being more included in family activities in our home”. Overall, most participants expressed the benefits of having more time to spend with their companion dog.

#### 3.2.2. Strained

The very small number of participants who reported increased time with their dog resulted in straining the relationship (3.2%), explained the strain came from having to negotiate multiple roles and responsibilities and encountering undesirable dog behaviors. For example, one participant noted: “Working from home means that I want to spend time with the dogs but I can’t because I have to work. The dogs are probably wondering why I’m not paying them more attention when I’m home”. One parent explained, “I have a toddler and they are annoying each other and annoying me by having to manage them”.

#### 3.2.3. Both Strengthened and Strained

Increased time together and the resultant increased bonds between humans and their canine companions came as a double-edged sword; on the one-hand, attunement and love increased, while on the other hand, these close connections resulted in increased vulnerability for separation anxiety. One-fifth of the participants stated that the increased time at home with their canine companions resulted in mixed feelings about the strength of their relationship. This group of individuals felt that more time together resulted in both the strengthening and straining of their relationship with their dog. Similar to previous responses, reflections of the ways in which relationships improved included more quality time, increased playtime and training time, and providing love and comfort. The strains centered around activity restrictions, dog behaviors, and worries about the future. An example of these mixed feelings appeared as follows:

“Dogs definitely like having us here and like to cuddle/be close. But their bad habits are extra annoying because we are home all the time and I feel they are also acting out a bit from boredom since we don’t go to the places we would normally.”

With respect to worries about the future, participants most commonly worried about how their dog would respond to them having to return to former work settings. For example: “They now do everything with me and I love it. I think it will strain when I go back to work. They will be depressed”.

### 3.3. Q3. Additional Thoughts about How Covid-19 Has Impacted Your Relationship with Your Dog

The final two open-ended questions aimed to gather additional thoughts and feelings from participants about the ways in which Covid-19 impacted their relationship with their canine companions. The broad nature of the questions enabled respondents to further reflect on previously mentioned themes and also offer new or nuanced representation of themes.

#### 3.3.1. More Quality Time/Enhanced Bond

In the first of these questions, the participants were asked to reflect on additional ways that their relationship with their dog has been impacted since the onset of Covid-19. The majority of responses revealed the positive ramifications of Covid-19 on human-animal bonds. For example, the most common themes focused on the benefit of more quality time in developing a sense of an enhanced bond with their dog. Members of the family who regularly worked away from home assumed new roles in relationship to their dogs and had time to nurture stronger bonds. As portrayed by one spouse, “My husband always works outside of the home but he’s home now working. One of my dogs has taken up a post at my husband’s side, all day, every day. I think he feels much closer to the pets now”. Statements of enhanced bond appeared in descriptions of increased connection, attachment, attunement, and love. As social distancing restrictions created a sense of isolation, dogs offered friendship that, for some, paralleled human bonds. As reflected by a participant, “Having a companion during this uncertain time, when I was unable to see my friends/family, has been a God-send”. While participants searched for meaning in the uncertainty of Covid-19, they discovered that the pandemic opened opportunities to cultivate enhanced bonds with their dogs and reduce their sense of isolation.

#### 3.3.2. Worries about Dog’s Well-Being

In addition, high among participants’ concerns were their worries about dog’s well-being. These concerns appeared as worries about access to food, veterinary care, and a newly emerging expression of concern about their dog’s emotional, physical, and social well-being since the onset of Covid-19. Fear resonated throughout responses about the ways in which participants’ emotional presence may negatively influence their canine companions’ emotional health. Due to the increased quality time and augmented attunement and intuition, dogs were observed as carrying the weight of their guardian’s stress. For example, “I think my older dog feels my anxiety and that’s upsetting me. I don’t want her feeling my anxiety so I’m doing my best to play and give her affection”. Participants additionally expressed concern that their work stress and pandemic-related anxiety was rubbing off on their dogs and appearing in their behaviors. One owner proclaimed, “My anxiety related to my work is felt by my dog and exacerbates her anxiety. Her behavior declines as a result and this stresses me more, so it’s a vicious circle”. Responses also echoed stress about not being able to access specialty dog foods or veterinary care. Other initial Covid-19 physical concerns included worry about dogs getting Covid-19, as one participant shared, “I don’t kiss her forehead anymore”. Expressions of worries about dog’s social well-being also centered around their restrictions in being able to carry out therapy dog activities. Participants shared stress in knowing that their dogs were not receiving physical touch, being able to work in their fulfilling roles, or achieve the mental stimulation they typically received from their social activities. One therapy dog owner explained:

“She is a therapy dog and a Crisis Response Dog. We travel together to give comfort to those in need, whether special kids or people in hospitals or survivors of mass shootings. She only has me and my husband to care for now so I have had to think of things to keep her busy. She’s used to being on the go.”

In their roles as caregivers, dog owners expressed significant worry in meeting their dog’s emotional, physical, and social needs and attempted to protect their dogs from the additional stresses resulting from Covid-19.

### 3.4. Q4. Covid-19 Impact on Feelings about Living with a Dog

The last question allowed participants to provide final reflections about living with a dog during Covid-19. Responses resoundingly portrayed gratitude for the ways in which canine companions eased the burdens of the pandemic. Themes that rose to the top among participants’ responses included gratitude, couldn’t live without a dog, dog is like family, and improves mental health. The following paragraphs characterize participants’ feelings about living with a dog during the pandemic.

#### 3.4.1. Gratitude

When given the opportunity to share any thoughts or feelings about their canine companions’ presence during Covid-19, more than half of participants expressed gratitude. Participants drew support from the presence of and interactions with their dogs. For example, they incorporated descriptive words about their dogs such as, “joy”, “sanity”, “friendships”, and “a sense of purpose”. For those that live alone, their canine companions appeared as lifesavers, “I was just talking to friends about how glad I am to have a dog right now, and how lonely it would be to be so isolated from people physically without my dog”. Although caring for a dog during the pandemic added additional stressors in navigating new roles, and providing enough exercise and training activities, most people could not imagine spending the lockdown time of the pandemic without their canine companions. In time, most owners adapted to the initial stressors and acknowledged their irreplaceable relationships with their dogs:

“I suspect [the dogs] sensed the increased stress in the household - they were certainly more attentive and would actively seek out physical contact. We seem to have adjusted to a new normal now with a lower current level of stress in this "holding pattern" time. I found the contact-seeking helpful, pleasurable and distracting from current events and appreciate my dogs more as a result.”

A common expression of gratitude focused on the value of having a canine partner to share the emotional and social roller coaster rides of the pandemic: “I couldn’t have asked for a better quarantine partner!!

#### 3.4.2. Couldn’t Live without a Dog/Dog Is Like a Family Member

The challenges resulting from Covid-19 affirmed for many individuals the instrumental roles that their canine companions play in their lives. Many participants proclaimed that they couldn’t live without their dog. The following sentiment resonated throughout responses: “My plan is to never be without a dog(s). I hope I’m never in a position, e.g., going to a nursing home where I couldn’t have them. Dogs are a very, very, very big and important part of my life”. Others reflected on the invaluable role that their dogs play in providing companionship and comfort: “I can’t imagine going through something as scary and isolating as this pandemic without a dog. I am a widow living with my adult daughter and her husband. They are wonderful to have here but I spend every minute with my dogs while I am home. They are truly constant companions”. The enhanced bonds fostered during Covid-19 and time spent caring for and playing with their dogs also left some people describing their dogs as family members. For example, one participant shared that their dog has become “more of a family member in my eyes”.

#### 3.4.3. Improves Mental Health

Among the reasons that participants expressed gratitude for their canine companions was the way in which they buffered the distress that they experienced from isolation, grief, loss, and unending uncertainty. As explained by a participant, “Having her in my home during this time of isolation has really helped me stay mentally well also because she gives me purpose and helps me feel less alone”. Caring for a canine companion appeared to foster a sense of control for instance, “Spending time with my dogs helps keep me grounded in the present and gives me other creatures to focus my energy on instead of worrying about things I cannot control”. Daily routines with canine companions also helped participants to live in the moment and appreciate the simple joys in life. This perspective was explained, “Dogs are wonderful especially in unpredictable times. They bring comfort and therapeutic mindfulness to help with stress and anxiety”. In summary, participants felt immense gratitude for the numerous benefits they garnered from their relationships with their canine companions.

## 4. Discussion

The thematic results found in this study highlight the strong connections between humans and their dogs and the role this bond played for pet owners during the initial stages of the pandemic. Despite the stressor of a global pandemic, the striking majority of respondents (76.8%) expressed that their dog reduced their level of stress and provided more positive than negative experiences. Having a dog to care for and focus upon also distracted them from their greatest fears and worries and were seen as a calming presence that facilitated groundedness in the moment. At the same time, dogs consistently enabled moments of joy and laughter, supporting positive emotions during stress. Having a canine companion also appears to have had a protective impact on mental and physical health through added daily structure and exercise. A recent study that assessed the role between companion animals and their humans during Covid-19, found that companion animal ownership was related to better mental health scores [41]. In the current study, spending time with their dog appeared to greatly diminish participants’ sense of isolation and loneliness. This suggests that relationships with dogs may offer benefits similar to human relationships and can potentially serve as surrogates in times of duress or loneliness.

Spending more time with their dogs, for the majority of respondents, appears to have strengthened the human–companion animal bond and cultivated openings for quality interactions. Participants noted that prior to Covid-19, their busy lives limited the amount of time they could spend with their dogs. Due to Covid-19 restrictions, they were able to reap the benefits of quality time with their dog through physical interactions, play, training, and the opportunity to care for them. In their roles as caregivers, participants cherished additional quality time to nurture the affection, love, and companionship they shared. This was especially true for people with elderly or sick dogs. More time also seemed to enhance participants’ experiences of harmony with their dog, increasing a sense of a reciprocal human–animal empathic connection and attunement.

The responses to these open-ended questions suggest that the participants were able to use the survey as a way to express their feelings in an anonymous, safe, way. When given the space to freely comment upon anything regarding their dog during the pandemic, respondents underscored the importance of their human/animal connection. Gratitude was a fundamental theme, and it is clear that dogs provided people with a sense of purpose. Consistent with previous research regarding the human and animal bond [23,24], dogs were seen as a support for mental health, as their canine companions offered not only a sense of connection, but also structure and routine. Nieforth and O’Haire [42] note that “individuals may perceive that their pets help to manage their uncertainty because caring for the pet remains a consistent routine in a time in which not much else is consistent or predictable.” (p. 246). For the most part, participants reported that their dog was an intrinsic part of their family, and they could not imagine their lives without them. If ever there was a time for the intrinsically social and playful characteristics of dogs to shine, the pandemic truly brought forth their most positive qualities.

From the onset of Covid-19, daily lives changed in unprecedented ways, leaving many individuals scrambling to find food, shelter, work, and a sense of normalcy. For a small subset of respondents, dog companionship compounded these stressors. More time with their canine companion also meant shifting family interactions with their dog, and the subsequent fallout, such as negotiating new relationships. Furthermore, the added responsibility of taking care of a living creature highlighted worries about the future. Strikingly, these worries were not about the participants themselves, but rather, how the pandemic could negatively impact their dog. At the same time, participants were reticent to note something negative about their relationship with their dog, no matter how stressful, without qualifying the statement with something positive. This orientation seems to once again speak to the dog owner’s predominant desire, above all else, to keep their dogs safe, healthy, and thriving.

The primary limitation of this study comes from the self-selection process. This sample consisted of US dog owners, primarily female (88.3%), who have access to the Internet, so care should be taken when generalizing to other populations. Additionally, the survey did not include questions about race, ethnicity, or socio-economic status, which are important variables for future studies, especially around the impact of this pandemic. Another limitation of this study involves its cross-sectional design. This study captures a moment in time, right at the beginning of the Covid-19 epidemic, when restrictions were just put in place. Thus, it is not possible to know how experiences have changed over time as the pandemic continues. Future research could explore more longitudinal issues, post-Covid-19, especially regarding the dog-human bond and its continuing impact on psychosocial functioning.

## 5. Conclusions

The world may be changed forever, with far fewer people going back to traditional work settings, and considerably fewer opportunities for social interactions than there were in the past. Thus, people may continue to feel a sense of disconnect and isolation, especially if they live by themselves. This study furthers the work of Nieforth and O’Haire [42], demonstrating how “People may turn to their pets as a source of contact comfort”, a fact that could be particularly important during the Covid-19 pandemic for individuals who are living alone or are physically isolated from loved ones (p. 246). The results of this qualitative study support the growing body of literature regarding the importance of people’s relationships with their dogs [28].

Many people are spending more time with their dogs now than prior to Covid-19. It is hoped that this positive impact of Covid-19 does not change as people return to some type of new normal. Policies that support dog ownership would be beneficial, as research has continually shown both the physical and mental health benefits of having a dog [23] as well as income and full-time employment being associated with an increased likelihood of dog ownership [43]. As shown in the current study, dogs can reinforce positive behaviors such as exercise, play, routine, and structure. Dogs are thought of as family members and many people express concerns about their dog before they consider their own needs. Gratitude regarding their dog was a dominant theme emanating from the participants, and empirical studies have demonstrated that gratitude can reduce negative mental health symptoms [44,45]. Through the uncertainties, fears, and losses resulting from the pandemic, canine companions offer meaning, comfort, and a hopeful distraction from the unsettling feelings of our daily lives.

## Figures and Tables

**Table 1:** Sample sociodemographics (N = 956).

Category	N (%)	M (SD)	Range
Gender			
Female	3610 (88.3)		
Male	443 (10.8)		
Non-binary	34 (0.8)		
No answer	18 (0.4)		
Country			
United States	3313 (80.7)		
Canada	355 (8.7)		
United Kingdom	177 (4.3)		
Australia	64 (1.6)		
Other	194 (4.7)		
Age		48.06 (14.27)	18–92
Under 30	558 (13.8)		
30–39	772 (19.0)		
40–49	790 (19.5)		
50–59	966 (23.8)		
60 and older	972 (24.0)		
Adults living in the home			
One	872 (21.2)		
Two	2533 (61.7)		
Other	700 (17.0)		
Children Living in the home			
None	3305 (80.5)		
One	423 (10.3)		
Other	377 (9.1)		
Dogs living in the home			
One	2028 (49.4)		
Two	1286 (31.3)		
Three	479 (11.7)		
Other	312 (7.5)		
Restriction level			
Non-essential businesses closed; ordered to stay at home	3197 (77.9)		
Non-essential businesses closed; not ordered to stay at home	724 (17.5)		
Some stores/businesses and restaurants closed	151 (3.7)		
No current restrictions	17 (0.4)		
Other	16 (0.4)		

## Data Availability

Data available by contacting the corresponding author.
